# Effects of Transapical Transcatheter Mitral Valve Implantation

**DOI:** 10.3389/fcvm.2021.633369

**Published:** 2021-06-11

**Authors:** Ming-Chon Hsiung, Wei-Hsian Yin, Yung-Tsai Lee, Tien-Ping Tsao, Kuo-Chen Lee, Kuan-Chih Huang, Pei-En Chen, Wei-Hsuan Chiang, Tao-Hsin Tung, Jeng Wei

**Affiliations:** ^1^Heart Center, Cheng Hsin General Hospital, Taipei, Taiwan; ^2^Faculty of Medicine, National Yang Ming University, Taipei, Taiwan; ^3^School of Medicine, Institute of Microbiology and Immunology, National Yang Ming University, Taipei, Taiwan; ^4^Faculty of Medicine, National Defense Medical Center, Taipei, Taiwan; ^5^Institute of Health Policy and Management, National Taiwan University, Taipei, Taiwan; ^6^Taiwan Association of Health Industry Management and Development, Taipei, Taiwan; ^7^Department of Medical Research and Education, Cheng Hsin General Hospital, Taipei, Taiwan

**Keywords:** mitral valve implantation, surgical redo mitral valve replacement, cardiac surgery, heart surgery, cardiovascular

## Abstract

**Purpose:** In this study, transapical transcatheter mitral valve-in-valve implantation (TAMVI) was compared with surgical redo mitral valve replacement (SRMVR) in terms of clinical outcomes.

**Methods:** We retrospectively identified patients with degenerated mitral bioprosthesis or failed annuloplasty rings who underwent redo SRMVR or TAMVI at our medical center. Clinical outcomes were based on echocardiography results.

**Results:** We retrospectively identified patients with symptomatic mitral bioprosthetic valve dysfunction (*n* = 58) and failed annuloplasty rings (*n* = 14) who underwent redo SRMVR (*n* = 36) or TAMVI (*n* = 36). The Society of Thoracic Surgeons Predicted Risk of Mortality scores were higher in the TAMVI group (median: 9.52) than in the SRMVR group (median: 5.59) (*p*-value = 0.02). TAMVI patients were more severe in New York Heart Association (*p*-value = 0.04). The total procedure time (skin to skin) and length of stay after procedures were significantly shorter in the TAMVI group, and no significant difference in mortality was noted after adjustment for confounding factors (*p*-value = 0.11). The overall mean mitral valve pressure gradient was lower in the TAMVI group than in the SRMVR group at 24 months (*p* < 0.01). Both groups presented a decrease in the severity of mitral and tricuspid regurgitation at 3–24 months.

**Conclusions:** In conclusion, the statistical analysis is still not robust enough to make a claim that TAMVI is an appropriate alternative. The outcome of the patient appears only to be related to the patient's pre-operative STS score. Additional multi-center, longitudinal studies are warranted to adequately assess the effect of TAMVI.

## Introduction

Over the past two decades, transcatheter aortic valve implantation (TAVI) has been established as a viable alternative treatment to deal with severe aortic stenosis in patients at risk of open-heart surgery. This procedure has also been extended to patients facing low-to-intermediate operative risk. This shift has been prompted by recent studies suggesting that TAVI provides survival benefits for high-risk and intermediate-risk patients (1). Mitral valve disease is the most common valvular disease in developed countries. There has been a notable shift away from mechanical valves toward bioprosthesis valves, despite their finite longevity. Recurrent mitral regurgitation (MR) is frequently encountered after mitral valve repair, particularly in cases of ischemic MR (2). However, reoperation imposes high risks among the aged and those with multiple comorbidities. Transapical transcatheter mitral valve-in-valve implantation (TAMVI) is now regarded as a promising alternative treatment for patients with degenerated bioprosthesis or failed annuloplasty (3). Most cases of TAMVI were reported in large registry and were done in some referral centers. The surgery was only reported with limited cases in Asian patients (4, 5). There have been a relatively small number of reports pertaining to the clinical outcomes and echocardiographic findings following TAMVI or surgical redo mitral valve replacement (SRMVR) for degenerated bioprosthetic valve or failed annuloplasty rings. In this study, we sought to determine whether TAMVI could achieve outcomes on par with those of SRMVR at 3, 6, 12, and 24 months after the procedure in terms of mortality rates and echocardiographic findings.

## Methods and Materials

### Study Subjects and Data Collections

**Figure 1:**
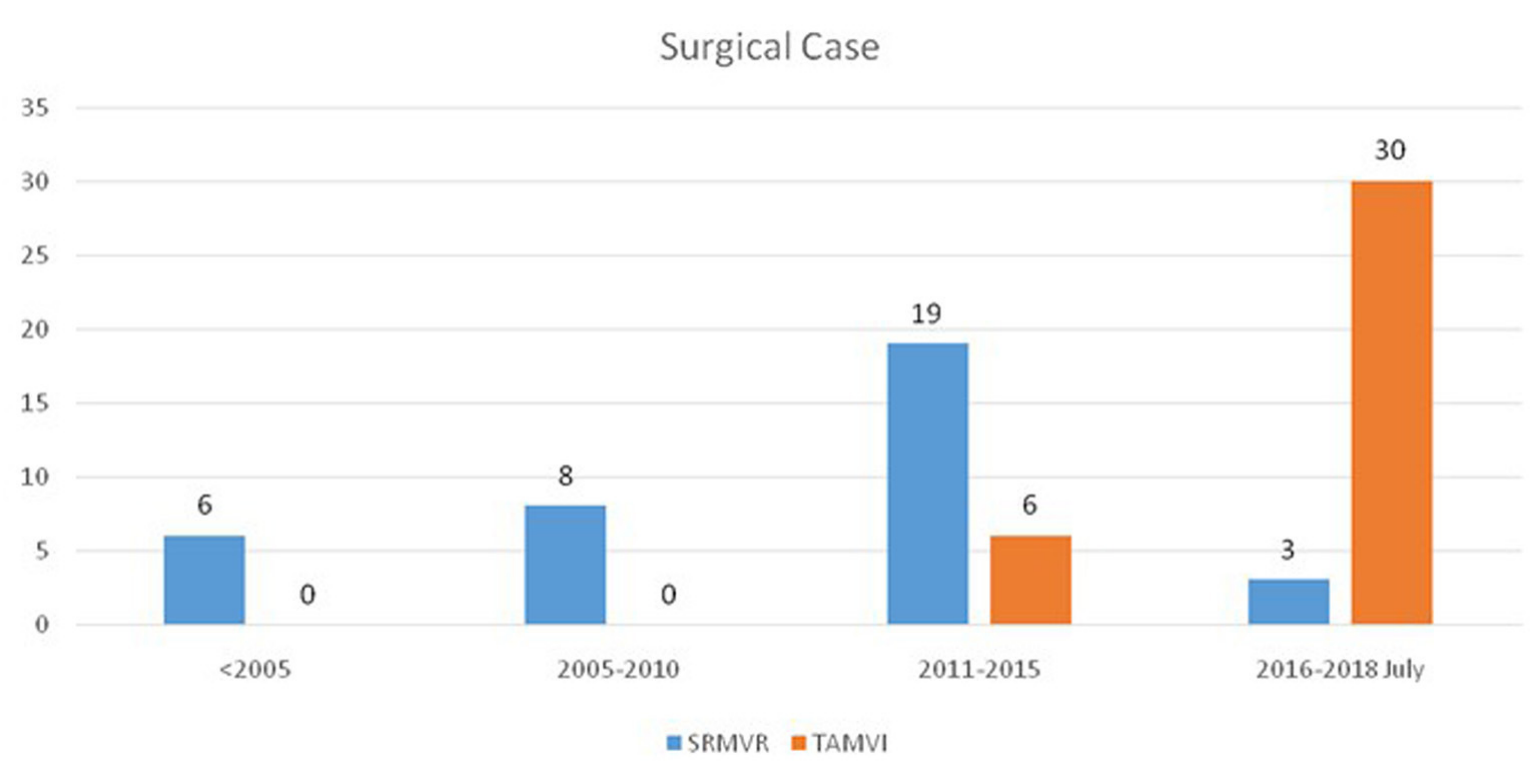
Change of the number of TAMVI compared with SRMVR in the study hospital (*n* = 72).

We retrospectively identified patients who underwent SRMVR or TAMVI for degenerated mitral bioprosthesis or failed annuloplasty rings at our medical center between 1998 and July 2018. Note that the TAMVI procedure was not performed until 2014 (Figure 1). In the SRMVR group, myocardial protection was achieved via aortic clamping and antegrade cardioplegia. The left atrium was approached via Waterson's groove or trans-septal (in cases where tricuspid intervention was required). Following replacement, the left atrium was closed and warmed cardioplegia was delivered prior to removal of the aortic cross clamp. Once the heart started beating and all vital signs were satisfactory, cardio pulmonary bypass (CPB) was weaned off and the sternum was closed. In the TAMVI group, all procedures were conducted using the transapical approach. All procedures were performed using general anesthesia under 2D and 3D trans-esophageal echocardiography guidance (TEE). The left ventricle apex was guided via Transthoracic Echo and agitated normal saline (2 cc) was injected into the left ventricle using a fine needle, whereupon a left anterior thoracotomy was performed through the fifth or sixth intercostal space. A guidewire was advanced through two 3-0 polypropylene purse string surfaces reinforced with Teflon pledget, across the malfunctioning bioprosthesis into the pulmonary vein. This procedure was performed solely under 2D or 3D TEE with no iodinated contrast. A stiffer guidewire (Amplatz Extra stiff; Cook Inc., Bloomington, IN) was then introduced using a Judkins catheter for protection. An Edwards Sapien, Sapien XT, Sapien S3 (Edwards Lifesciences, Irvine, CA), or Lotus (Boston Scientific) was delivered using the standard delivery system (Ascendra, Ascendra +, Commander; Edwards Life sciences) during rapid ventricular pacing 130–180 BPM.

All of the patients included in this study had degenerated mitral bioprosthesis or failed annuloplasty rings. The treatment method was determined by a multi-disciplinary heart team in accordance with the anticipated risk and anatomical specifics of each patient. Echocardiographic parameters were reported in accordance with the guidelines defined by the American Society of Echocardiography and the Mitral Valve Academic Research Consortium (MVARC) consensus document (6–8). All transcatheter procedures were performed using a balloon-expandable transcatheter heart valve (Edward Sapien 3, XT) or heart valve (Lotus, Boston Scientific), which was manually implanted via transapical access, as previous described (9–11). The access route and valve size were based on procedural echocardiographic findings and multidetector computed tomography (MDCT). The exclusion criteria included active endocarditis, cases requiring concomitant procedures for aortic disease or coronary artery disease. We also excluded the patients with a measured new-left ventricular outflow tract area (new-LVOT) <150 cm^2^ at end-systolic phase and aortic-mitral angle <135° (12).

Sample data included detailed clinical information on 72 patients, 36 of whom underwent TAMVI (50%) and 36 of whom underwent SRMVR with cardiopulmonary bypass (50%). Valve type was determined prior to the procedure, whereas valve size was determined during the procedure by calculation with proprietary valve size. None of the patients who needed concomitant aortic valve replacement or had prosthetic valves developed mitral valve endocarditis (in the time period).

In this study, all patient information, outcomes, and complications were derived from electronic medical records. This study was approved by the Regional Ethical Review Board and all patients provided written informed consent.

### Statistical Analysis

Continuous variables were reported as mean ± SD or median (range) and tested using a two-sample independent *t*-test or Mann-Whitney *U* test, respectively. Categorical variables were examined using the chi-square test or fisher's exact test. A repeated measures analysis of variance and McNemar's test were performed to clarify the results of the echocardiographic findings between the SRMVR and TAMVI groups. Survival curves were analyzed using the Kaplan-Meier method and compared using the log-rank test. A two-sided *p*-value of <0.05 was considered statistically significant. All statistical analysis was performed using IBM SPSS statistics, version 20 (IBM, Armonk, NY).

In addition, the estimates of screening for important variables using univariable cox regression and including the significant variables is not the ideal way to approach model construction. Because subjects in the TAMVI and SRMVR groups likely differ for confounding factors and differences in outcomes could reflect differences in baseline conditions rather than a real treatment effects, inverse probability of treatment weighting (IPTW) was used to compare the two treatments (13).

## Results

In this study, a total of 72 redo mitral valve replacement patients (TAMVI: *n* = 36; SRMVR: *n* = 36) met the inclusion criteria, including patient characteristics and operative data. Study-patients TAMVI (30/36 = 83.3%) exhibited a more-pronounced proportion after 2016 than SRMVR (3/36 = 8.3%) (Figure 1). Table 1 lists the baseline characteristics of the study population. The age difference is not statistically significant between patients who underwent TAMVI implantation and underwent SRMVR (65.28 ± 13.89 vs. 59.61 ± 15.92, *p* = 0.11). In addition to higher median STS Predicted Risk of Mortality scores (9.52 [range: 1.16–78.41] vs. 5.59 [range: 1.12–44.15], *p* = 0.02 for Mann-Whitney *U* test), TAMVI patients are also more likely to have liver disease, CAD, and severe heart failure. Indications for reoperation included recurrent severe mitral regurgitation in 16 patients (22.2%), isolated mitral stenosis in 44 (61.1%), and mixed mitral regurgitation and stenosis in 12 (16.7%).

**Table 1:** Baseline clinical characteristics between SRMVR and TAMVI groups (*n* = 72).

	**SRMVR (*n* = 36)**	**TAMVI (*n* = 36)**	***P*-value**
	**Mean ± SD or** ***n* (%)**	**Mean ± SD or** ***n* (%)**	
Age (years)	59.61 ± 15.92	65.28 ± 13.89	0.11
BSA (m^2^)	1.58 ± 0.20	1.57 ± 0.18	0.91
STS score	Median: 5.59; range (1.12–44.15)	Median: 9.52; range (1.16–78.41)	0.02
Male	13 (36.1)	16 (44.4)	0.63
Diabetes	7 (19.4)	8 (22.2)	1.00
Dyslipidemia	18 (50.0)	15 (41.7)	0.64
AKD	0 (0.0)	2 (5.6)	–
CKD	11 (30.6)	8 (22.2)	0.59
H/D	2 (5.6)	4 (11.1)	0.67
Lung disease	7 (19.4)	10 (27.8)	0.58
Liver disease	1 (2.8)	6 (16.7)	0.11
CVA	2 (5.6)	6 (16.7)	0.26
CAD	6 (16.7)	16 (44.4)	0.02
PVD	1 (2.8)	2 (5.6)	1.00
Endocarditis history	7 (19.4)	4 (11.1)	0.51
Old MI	1 (2.8)	0 (0.0)	–
PCI	2 (5.6)	6 (16.7)	0.26
CABG	3 (8.3)	8 (22.2)	0.19
AF	17 (47.2)	20 (55.6)	0.64
PPM	3 (8.3)	5 (13.9)	0.71
LA thrombus	4 (11.1)	2 (5.6)	0.67
Arrhythmia	9 (25.0)	4 (11.1)	1.00
AKD + H/D	1 (2.8)	1 (2.8)	1.00
Indications			0.17
Mitral regurgitation	6 (16.7)	10 (27.8)	
Isolated mitral stenosis	26 (72.2)	18 (50.0)	
Mixed mitral regurgitation and stenosis	4 (11.1)	8 (22.2)	
NYHA			0.04
2	12 (33.3)	6 (16.7)	
3	19 (52.8)	16 (44.4)	
4	5 (13.9)	14 (38.9)	
Previous MV replacement	25 (69.4)	33 (91.7)	0.04
Previous MV repair	11 (30.6)	3 (8.3)	0.04
Previous AV replacement	5 (13.9)	10 (28.6)	0.16
Previous TV repair	14 (38.9)	17 (48.6)	0.48

*BSA, body surface area; STS, society of thoracic surgeons; AKD, acute kidney disease; CKD, chronic kidney disease; H/D, hemodialysis; CVA, cerebrovascular accident; CAD, coronary artery disease; PVD, peripheral artery disease; MI, myocardial infarction; PCI, percutaneous coronary intervention; CABG, coronary artery bypass graft; AF, atrial fibrillation; PPM, permanent pacemaker; LA, left atrium; NYHA, New York Heart Association; MV, mitral valve; AV, aortic valve; TV, tricuspid valve*.

Procedural details for surgical intervention are summarized in the Table 2. The main proportion of replacing valve type were ST-JUDE (44.4%) and Edwards Sapien XT (63.9%) in the SRMVR and TAMVI groups, respectively. Table 3 further indicates the comparisons of in-hospital outcomes between SRMVR and TAMVI groups. To compare the TAMVI groups, SRMVR groups had longer total procedure time (520.97 ± 85.56 min vs. 177.50 ± 115.86 min, *p* < 0.001) and longer length of stay after procedures (28.47 ± 12.15 min vs. 21.89 ± 8.60 min, *p* = 0.01). There was one case of stroke after SRMVR but no cases of stroke after TAMVI. There were no indications of left ventricular outflow tract (LVOT) obstruction in either group. There were no significant differences between the two groups in terms of bleeding or arrhythmia. In addition, the mean cardiopulmonary bypass duration was 181.97 ± 63.20 min, whereas the aortic clamp duration was 153.39 ± 66.96 min. Tricuspid valve repair was performed as a concomitant procedure in six cases and ablation for atrial fibrillation in five cases, based on the judgment of the operating surgeon.

**Table 2:** Procedure details in SRMVR or TAMVI approaches.

**SRMVR**	***n* (%)**
**APPROACH**	
Median sternotomy	36 (100.0)
**REPLACING VALVE TYPE**	
Edwards Sapien	10 (27.8)
ST-JUDE	16 (44.4)
ON-X	5 (13.9)
Medtronic-Hancock	2 (5.6)
SORIN BICARBON	1 (2.8)
C-E perimount magna mitral ease	1 (2.8)
Mosaic tissue valve	1 (2.8)
**CONCOMITANT SURGICAL PROCEDURES**	
Tricuspid valve repair	6 (16.7)
Ablation for atrial fibrillation	5 (13.9)
**TAMVI**	***n*** **(%)**
**APPROACH**	
Transapical	36 (100.0)
**REPLACING VALVE TYPE**	
Edwards Sapien	6 (16.7)
Edwards Sapien XT	23 (63.9)
Boston Scientific Lotus	7 (19.4)

**Table 3:** The comparisons of in-hospital outcomes between SRMVR and TAMVI groups (*n* = 72).

	**SRMVR (*n* = 36)**	**TAMVI (*n* = 36)**	***P*-value**
	**Mean ± SD or *n* (%)**	**Mean ± SD or *n* (%)**	
Replacing valve inner diameter (mm)	28.17 ± 1.88	27.92 ± 1.52	0.54
Urgent procedure	7 (19.4)	2 (5.6)	0.08
Total procedure time (min)	520.97 ± 85.56	177.50 ± 115.86	<0.001
CPB time (min)	181.97 ± 63.20	–	–
Cross-clamp time (min)	153.39 ± 66.96	–	–
Fluoroscopy time (min)	–	10.00 ± 2.00	–
Amount of contrast (ml)	–	0.00	–
IABP utilization (intra-/post-procedure)	3 (8.3)	0 (0.0)	1.00
Total ICU stay (days)	6.56 ± 5.47	4.47 ± 5.86	0.12
Length of stay after procedures (days)	28.47 ± 12.15	21.89 ± 8.60	0.01
Delayed LV apical pseudoaneurysm	0 (0.0)	1 (2.8)	1.00
**CLINICAL OUTCOMES**			
Post-PPM	2 (5.6)	1 (2.8)	0.23
In-hospital death	1 (2.8)	0 (0.0)	1.00
Minor complication	2 (5.6)	0 (0.0)	1.00
Bleeding complication	3 (8.3)	1 (2.8)	0.30
Stroke	1 (2.8)	0 (0.0)	–
Arrhythmia	9 (25.0)	4 (11.1)	0.13
LVOT obstruction	0 (0.0)	0 (0.0)	–

*PPM, permanent pacemaker*.

Table 4 indicates the no statistical significantly for the comparisons of in-hospital outcomes between the two TAVI products. As Table 5 shows, only borderline significant was found in total ICU stay between failed annuloplasty ring and non-failed annuloplasty (12.33 ± 13.65 vs. 3.76 ± 4.43 days, *p* = 0.05 for Mann-Whitney U test). In addition, in the mitral valve in ring (TMVIR) groups, we did three patients, all patients under TAMVI implantation with two Edwards Sapien XT and one Boston Scientific Lotus valve. As shown in Table 6, two of these patients were event-free; however, one of the patients expired 21-day post-operation due to multiple organ failure (STS score = 73.665).

**Table 4:** The comparisons of in-hospital outcomes between the two TAVI products (*n* = 36).

	**Boston Scientific Lotus (*n* = 7)**	**Edward Sapien (*n* = 29)**	***P*-value**
	**Mean ± SD or *n* (%)**	**Mean ± SD or *n* (%)**	
Replacing valve inner diameter (mm)	27.29 ± 0.76	34.97 ± 37.35	0.17
Total procedure time (min)	155.71 ± 52.08	182.76 ± 126.69	0.70
Total ICU stay (days)	2.57 ± 2.37	4.93 ± 6.38	0.14
Length of stay after procedures (days)	25.71 ± 6.60	20.97 ± 8.86	0.06
**CLINICAL OUTCOMES**			
Post-PPM	0 (0)	5 (17.2)	–
Bleeding complication	0 (0)	1 (3.4)	–
Arrhythmia	0 (0)	4 (13.8)	–

*PPM, permanent pacemaker*.

**Table 5:** The results for value in ring compare to valve in prosthesis (*n* = 36).

	**Failed annuloplasty ring (*n* = 3)**	**Non-failed annuloplasty (*n* = 33)**	***P*-value**
	**Mean ± SD or *n* (%)**	**Mean ± SD or *n* (%)**	
Replacing valve inner diameter (mm)	27.00 ± 1.73	34.06 ± 35.03	0.27
Total procedure time (min)	148.33 ± 42.53	180.15 ± 120.34	0.91
Total ICU stay (days)	12.33 ± 13.65	3.76 ± 4.43	0.05
Length of stay after procedures (days)	24.67 ± 8.51	21.64 ± 8.69	0.51
**CLINICAL OUTCOMES**			
Post-PPM	0 (0.0)	5 (15.2)	–
Bleeding complication	1 (33.3)	0 (0.0)	–
Arrhythmia	0 (0.0)	4 (12.1)	–

*PPM, permanent pacemaker*.

**Table 6:** The details of patients with failed annuloplasty rings (*n* = 3).

**No**	**Age**	**Valve type**	**Baseline** ** STS score**	**Baseline** ** NYHA class**	**No. of prior** ** thoracotomies**	**Comorbidities**	**Clinical outcomes**
1	72	Boston Scientific Lotus	14.24	IV	1	History of mitral valve repaired, HTN, dyslipidemia, CKD, CAD, PCI, AF	Alive
2	81	Edwards Sapien XT	73.67	IV	1	History of mitral valve repaired, CKD, H/D, lung disease, CAD, PCI, CABG, PPM, bleeding complication	Expired (21days)
3	64	Edwards Sapien XT	5.25	III	1	History of Mitral Valve Repaired, HTN, CAD, AF	Alive

*HYN, hypertension; CKD, chronic kidney disease; CAD, coronary artery disease; PCI, percutaneous coronary intervention; AF, atrial fibrillation; H/D, hemodialysis; CABG, coronary artery bypass graft; PPM, permanent pacemaker; STS, society of thoracic surgeons; NYHA, New York Heart Association*.

**Figure 2:**
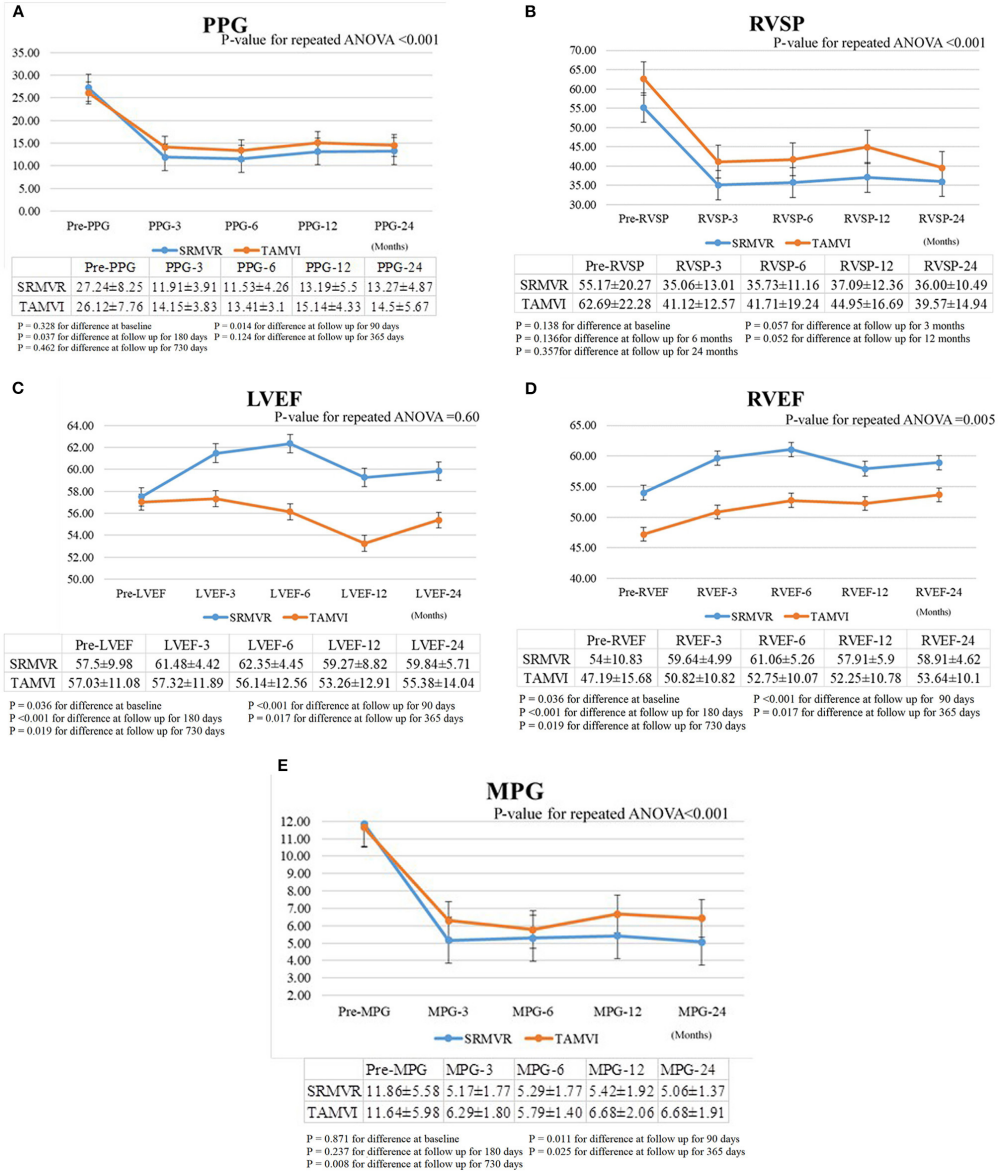
The comparison of mean pressure gradient (MPG) **(A)**, transmitral prosthesis gradient (PPG) **(B)**, right ventricular systolic pressure (RVSP) **(C)**, left ventricular ejection fraction (LVEF) **(D)**, right ventricular ejection fraction (RVEF) **(E)** between surgical redo mitral valve replacement (SRMVR) and transapical transcatheter mitral valve-in-valve implantation (TAMVI) (*n* = 72).

As shown in Figures 2A–D, hemodynamic results were deemed satisfactory based on a significant reduction in the mean pressure gradient, transmitral prosthesis gradient, and right ventricular systolic pressure for both SRMVR and TAMVI groups. The significant lower were found of these three hemodynamic results in the SRMVR groups than TAMVI groups (p-value for the repeated ANOVA < 0.001). Figure 2E shows that TAMVI groups had lower right ventricular ejection fraction than SRMVR groups during 24-month follow-up (*p* = 0.005).

**Figure 3:**
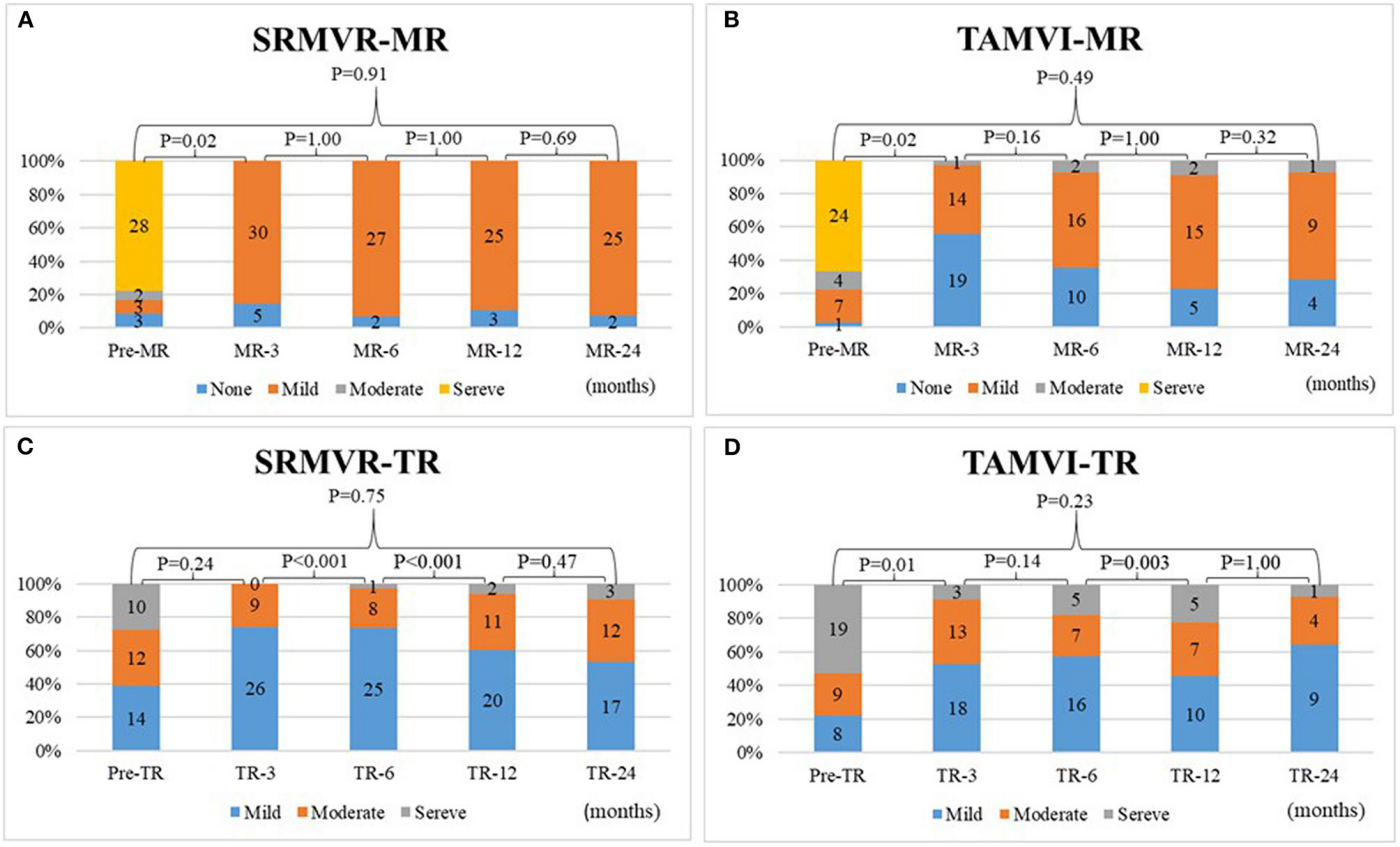
The comparison of degrees of mitral regurgitation **(A,B)** and tricuspid regurgitation **(C,D)** in surgical redo mitral valve replacement (SRMVR) and transapical transcatheter mitral valve-in-valve implantation (TAMVI) (*n* = 72).

Figure 3 shows that the degrees of mitral regurgitation significantly regress to mild or none only after 3 months in both SRMVR (3A) (*p* = 0.02) and TAMVI (3B) (*p* = 0.02) groups. No significant changes were found based on the multiple comparisons. The disparity of tricuspis regurgitation was found between SRMVR (3C) and TAMVI (3D) groups.

**Figure 4:**
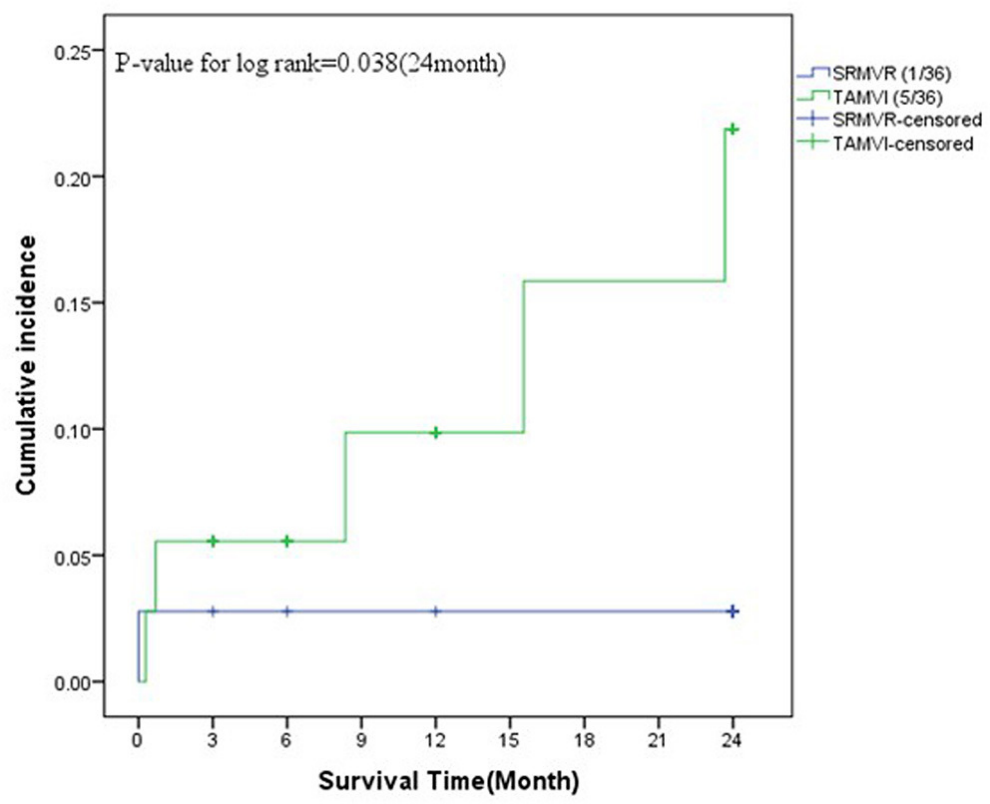
The survival analysis between surgical redo mitral valve replacement (SRMVR) and transapical transcatheter mitral valve-in-valve implantation (TAMVI) (*n* = 72).

As Figure 4 shows, in the SRMVR patients, the 3-, 6-, 12-, and 24-month cumulative mortality were 2.8%. In TAMVI patients, the 3-, 6-, 12-, and 24-month cumulative mortality were 5.6, 5.6, 9.8, and 21.9%, respectively. The statistical significance (*p* = 0.038) of procedure difference was found for cumulative survival.

The effect of independently associated risk factors upon all-cause mortality among patients after SRMVR or TAMVI surgery was examined using the multiple Cox regression models. As is depicted in Table 7, subsequent to adjustment for confounding factors and IPTW, STS score (Hazard ratio = 1.05, 95%CI: 1.01–1.09) appeared to be statistically significantly related to all-cause mortality.

**Table 7:** Multivariate analysis using Cox regression model of risk factors associated with the all-cause mortality after adjustment for inverse probability of treatment weighting (IPTW) among patients with SRMVR or TAMVI surgery (*n* = 72).

**Variables**	**All causes of death (yes vs. no)**				
	**β**	**SE**	***P*-value**	**Hazard ratio**	**95% confidence interval**
STS score	0.05	0.02	0.01	1.05	1.01–1.09
Operation (TAMVI vs. SRMVR)	2.91	1.74	0.09	18.41	0.61–553.92
Procedure time	0.004	0.003	0.21	1.004	0.998–1.011
IPTW	0.07	0.25	0.78	1.07	0.65–1.77

## Discussion

### Clinical Implications

Surgical valve replacement is the gold standard for patients with mitral valve disease or bioprosthesis failure. Nonetheless, recurrent MR is frequently encountered after mitral valve repair, particularly in the setting of functional MR (3). For most individuals, intervention provides functional improvement and increased survival exceeding that of clinical treatment (2, 14). However, many patients (particularly those with comorbidities) require reoperation, which brings with it an elevated risk of surgery-related complications (15–17). Since its introduction in 2009, transcatheter mitral valve-in-valve implantation has been increasingly adopted for the treatment of patients with malfunctioning bioprosthesis (18). Currently, there is a lack of data pertaining to TAMVI, and no direct comparison of TAMVI and SRMVR has been published in Asia. One study reported on 62 patients who underwent TAMVI and 59 patients who underwent SRMVR in three medical centers. They reported no difference between the two groups in terms of mortality at 12-month (19). Our results suggest that TAMVI could achieve results comparable to those of SRMVR in terms of 24-month all causes mortality due to this procedure introduces less surgical trauma and is less invasive (20, 21). Patients with poor left ventricular function were usually precluded SRMVR. Patients received SRMVR have higher risks of post-operative bleeding, longer ventilator usage time, and longer hospital stay (22, 23).

We acknowledge that our findings will have to be confirmed in subsequent research with a larger study population over a longer follow-up period. Note that our findings are also limited by the fact that the mean age in TAMVI group was not higher than in SRMVR group (*p* = 0.11), but the STS Predicted Risk of Mortality scores were higher. Note also that the SRMVR cohort included patients over a far longer period of time (from 1998 to July 2018). Since its introduction, the TAMVI technique has evolved in terms of planning (valve apps, MDCT and 2D, 3D echocardiography) and approach (from transapical), such that the procedure has become more effective, less invasive, and safer. Thus, our overall results may be influenced by difficulties experienced shortly after adoption; however, we were unable to excise the early cases due to the small number of patients in our sample.

Previous evidence-based studies reported mean gradient after TAMVI were 11.3 ± 5.2 to 5.5 ± 3.6 mmHg, 14.0 ± 6.5 to 4.7 ± 3.1 mmHg, and 6.3 ± 2.9 (immediate) to 7.3 ± 2.5 mmHg after 70, 130 days, and 1 year, respectively. The findings were consistent with our TAMVI results at 3 months from 11.64 ± 5.98 to 6.29 ± 1.80 mmHg at 3-month, 6.43 ± 1.91 mmHg at 24-month. This noted that the mean gradient after SRMVR includes mechanical and bioprosthetic valves (24–28). In addition, our results suggest that an elevated post-procedural mean gradient can still be a limitation after a transcatheter valve-in-valve procedure in both the aortic and mitral positions, but new techniques, such as transcatheter bioprosthetic valve fracture during TAMVI maybe a solution for patients with a small bioprosthetic valve (29–31).

There is always a risk of left ventricular outflow tract obstruction after TAMVI or SRMVR, due to interference from the surgical implant/TAMVI frame or paradoxical septal motion in patients with right ventricular volume overload following surgery. None of the patients in the current study presented LVOT obstruction after TAMVI (0%), most likely due to pre-procedural planning using 2D and 3D TEE and MDCT (32, 33).

The original idea for valve-in-ring procedures was proposed by Wilbring et al. (34). The most recent series was published by Urena et al. in which 30 Edward Sapien XT and Sapien 3 valves were implanted (35). Post-operative echocardiography results revealed hemodynamic outcomes. Note that valve sizing (and particularly determining the inner diameter) is essential to the success of valve-in-ring procedures; however, it remains the most challenging aspect of the procedure. We used the ring area provided by the manufacturer to estimate the internal diameter, under the assumption that the ring area and circumference would remain constant even after TAMVI implantation. Unfortunately, annuloplasty rings are semi-rigid, and a TAMVI is not capable of reshaping after implantation. Surprisingly, these gaps appear not to have any effect in implantations in terms of paravalvular leakage. We assume that surrounding valvular tissue or pannus formation seal these gaps. All valve-in-ring procedures in our series produced excellent hemodynamic results with good prosthesis function.

Concomitant surgical tricuspid repair is recommended for patients with more than mild tricuspid regurgitation at the time of mitral valve surgery, due to the fact that it does not increase the risk of operative mortality (36–38). Nonetheless, its effect on clinical long-term outcome remains an issue of controversy, despite acute echocardiographic improvement (39–41). Interestingly, in this study, for all SRMVR and TAMVI patients who did not undergo any tricuspid procedures (22 vs. 19), reductions of TR were similar (Figure 4) in 12-month mortality.

There are only limited cases in valve-in-ring, and mostly done by complete ring. The sizing guidance is according to the mitral VIV app developed by Bapat et al. incorporation with the technology company UBQO (42). We also performed the bench test according to the sizing chart of the app. Usually, oversizing 20% of the correspondent valve area by overfilling the balloon was done. All these valve-in-ring cases received surgical complete ring (case1: Edwards classic ring; case 2: Edwards Physio I ring; case 3: Sorin Memo 3D). In our bench test, the Edwards classic and Physio ring could be fitted by oversizing the implanted valve. However, Sorin Memo 3D remained some paravalvular space and resulted in paravalvular leakage.

Yoon et al. Reported in the TMVR registry only 80–90% success with relative lower success in the valve in ring group than our results (2). There are several important issues to success in our series. First, patients received pre-operative 4-D MSCT. The neo-LVOT, especially in mitral-valve-ring, was analyzed before surgery. If the neo-LVOT is <30% of original LVOT, we suggested patients should receive redo-surgery. Second, we performed all procedures from transapical access. We belief transapical access providing better coaxiality than trans-septal access. Besides, there are several cases received mitral procedure from trans-septal approach and bi-atrial approaches during first open heart surgery. It makes mitral valve-in-valve and valve-in-ring procedures from trans-septal approach more difficult. Currently, the procedure time of transapical mitral valve-in-valve and valve-in-ring procedures was <3 h. And there was only one case with apical pseudoaneurysm formation. Third, all patients received general anesthesia and transesophageal echocardiography. We belief additional TEE image providing neo-LVOT size and gradient. And also TEE monitoring the depth of the devices and prevent left atrial or left ventricle embolization.

We had a 58-year-old male patient who underwent TAMVI with a 26 mm Sapien 3 valve. After discharge, he remained symptom-free for a period of 3 weeks, at which point he began complaining of shortness of breath. Echocardiographic analysis revealed a large pseudoaneurysm in the left ventricular apex region. The patient subsequently underwent transcatheter closure of the pseudoaneurysm using an 18 mm Amplatzer atrial septal defect occluder (St. Jude Medical). As results indicated in previous mitral transcatheter studies, prescribing warfarin may prevent early valve thrombosis; however, there is no clear evidence to prevent the pseudoaneurysm in patients (25, 33). The Coumadin was routinely prescript in valve-in-valve, valve-in-ring, and valve-in-calcification cases. When patients have bleeding problems, the coumadin was stopped and used antiplatelets only. We believe anticoagulation was indicated in these cases due to most of these cases have atrial fibrillation. In additional to these reasons, trans-mitral blood flow is lower and higher residual trans-mitral pressure gradient in these cases.

In addition, Figures 3A,B indicates that vast majority of patients after surgical redo MVR appear to have mild regurgitation. The implanted Edwards mitral pericardial valve tend to have mild central valvular regurgitation. In our observation and literatures, this mild regurgitation did not have effect on short-term and long-term outcome (43, 44). We also found the Edwards Sapien valve has the same regurgitation esp. in previous generation of Sapien XT (45). This implies that the central regurgitation is a common finding related to Edwards bovine pericardial valve and does not effect short- and long-term durability of this bioprosthesis.

### Study Limitations

Although the main strength was that we tried to accommodate inherent selection biases with IPTW, the primary limitation of this study was the small number of patients, which may have limited the power to detect significant differences. The decision of whether to adopt TAMVI or SRMVR was made by the heart team in conjunction with the patient. It is likely that this approach introduced bias that could affect outcomes. We made our comparison as homogeneous as possible. However, there are still some differences that may influence the outcomes, including the incidence of new arrhythmias or other side-effects. Second, our inability to collect details related to the initial SRMVR procedures (many files were missing) may have affected the echocardiographic results and clinical outcomes. Third, fewer SRMVR patients underwent echocardiographic examinations at the time of discharge or during follow-up. The fact that we included patients who underwent SRMVR prior to the advent of TAMVI (80% of SRMVR procedures were performed between 1998 and 2013) meant that we had access to far more follow-up information for SRMVR patients than TAMVI patients. Finally, the perioperative mortality associated with redo mitral surgery is relatively lower than the reiterative mortality seen in the state of New York and Virginia (46, 47). The TAMVI mortality is also lower than that reported by Yoon et al. from the TMVR registry (2). Further long-term studies with a larger number of patients will be required to accurately assess the efficacy of TAMVI.

## Conclusions

In conclusion, this is a retrospective cohort study from Taiwan examining SRMVR in comparison with TAMVI. Transcatheter techniques will play a large role in the management of SVD in the future with the rapid increase in the use of bioprosthetic valves in younger and younger patients. The statistical analysis is still not robust enough to make a claim that TAMVI is an appropriate alternative. The outcome of the patient appears only to be related to the patient's pre-operative STS score. Equivalence has not been demonstrated, as a non-inferiority study would be required for such a claim to be made. The most that might be able to be said is that at our institution TAMVI has been a viable alternative in patients with adequate access and appropriate LVOT dimensions. For this reason, accumulating single institution reports are an important piece of the overall puzzle that will necessarily include larger scale examinations.

## Data Availability Statement

The data analyzed in this study is subject to the following licenses/restrictions: the database need permission from the hospital. Requests to access these datasets should be directed to jengwei@mac.com.

## Author Contributions

M-CH, W-HY, P-EC, T-HT, and JW conducted the study and drafted the manuscript. M-CH, P-EC, and T-HT participated in the design of the study and performed statistical analyses. Y-TL, T-PT, K-CL, K-CH, and W-HC conceived the study and participated in its design and coordination. All authors read and approved the final manuscript.

## Conflict of Interest

The authors declare that the research was conducted in the absence of any commercial or financial relationships that could be construed as a potential conflict of interest.

## Abbreviations

TAMVI: transapical transcatheter mitral valve-in-valve implantation
SRMVR: surgical redo mitral valve replacement
NYHA: New York Heart Association
MR: mitral regurgitation
CPB: cardio pulmonary bypass
TEE: trans-esophageal echocardiography
MVARC: Mitral Valve Academic Research Consortium
MDCT: multidetector computed tomography
IPTW: inverse probability of treatment weighting
LVOT: left ventricular outflow tract
TMVIR: the mitral valve in ring
MVARC: Mitral Valve Academic Research Consortium.
